# *L. Cucurbita pepo* Alleviates Chronic Unpredictable Mild Stress via Modulation of Apoptosis, Neurogenesis, and Gliosis in Rat Hippocampus

**DOI:** 10.1155/2021/6662649

**Published:** 2021-07-10

**Authors:** Nasra Ayuob, Soad Ali Shaker, Etedal Hawuit, Nouf saeed Al-Abbas, Nehad A. Shaer, Soad Al Jaouni, Mohamed R. Mahdi

**Affiliations:** ^1^Department of Medical Histology, Faculty of Medicine, Damietta University, Damietta, Egypt; ^2^Department of Histology, Faculty of Medicine, Assuit University, Egypt; ^3^Department of Anatomy, Faculty of Medicine, King Abdulaziz University, Saudi Arabia; ^4^Department of Biology, Faculty of Science, King Abdulaziz University, Jeddah, Saudi Arabia; ^5^Department of Biology, Jumum College University, Umm Alqura University, Makkah, Saudi Arabia; ^6^Department of Chemistry, Al-Leith College, Umm Alqura University, Makkah, Saudi Arabia; ^7^Department of Hematology/Pediatric Oncology and Yousef Abdul Latif Jameel Scientific Chair of Prophetic Medicine Application, Faculty of Medicine, King Abdulaziz University, Saudi Arabia; ^8^Department of Human Anatomy and Embryology, Faculty of Medicine, Mansoura University, Egypt

## Abstract

Pumpkin has received significant attention due to its nutritional compounds that have antioxidant, antifatigue, and anti-inflammatory effects. This study is aimed at assessing the antidepressant-like effect of *L. Cucurbita pepo*, sweet pumpkin, in an animal model of chronic unpredictable mild stress (CUMS) and investigating its effect on the histological structure of hippocampus compared to fluoxetine. Forty male albino rats assigned into the negative control, positive control (CUMS), and Flu-treated and pumpkin-treated groups (*n* = 10) were utilized in this study. Exposing rats to CUMS continued for 28 days, and treatments used were applied during the last 14 days of exposure. Behavioral, biochemical, and histopathological changes were assessed after 28 days. In this study, pumpkin significantly reduced the immobility time (*p* = 0.02), corticosterone (*p* < 0.001), TNF-*α*, IL-6 (*p* < 0.001), and malondialdehyde (*p* = 0.003), whereas it significantly increased the level of superoxide dismutase (SOD), catalase, and glutathione peroxidase (GPX) in the serum of rats exposed to CUMS. Pumpkin markedly relieved the degenerative and atrophic changes observed in the CA3 region and the dentate gyrus of the hippocampus. It significantly reduced caspase-3 and increased glial fibrillary acidic protein (GFAP) immunoexpression in the CA3 and DG. In conclusion, administration of pumpkin extract improved the behavioral, biochemical, and hippocampal pathological alternations induced in rats after exposure to CUMS in a comparable pattern to fluoxetine. This study highlighted the potential efficacy of pumpkin in alleviating depression disorder either alone or in conjugation with conventional antidepressant therapy.

## 1. Background

In modernized countries, regardless of the income level, there is an increment of psychological problems like depression [1]. Exposure to chronic external stress factors leads to the induction of oxidative and inflammatory pathways, and in susceptible individuals, they cause depression [2]. In animals, chronic stress induces apoptosis and neuroinflammation and inhibits neurogenesis in different brain regions [3, 4].

Hippocampus is very sensitive to stress hormones and antidepressant drugs [5]. Dentate gyrus (DG), CA1, and CA3 are distinct subregions of hippocampus, and each subregion has its physiological and pathological role in relation to depression behaviors and antidepressant effects [6]. It was reported that in patients with major depression, there was hippocampal atrophy as showed by brain radiograph [7]. This atrophy could be due to several factors including apoptosis [8]. Caspase-3 is a cysteine protease marker of apoptosis, and its activation by mitochondria is implicated in long-term depression [9]. This opens a promising path to understanding more about the molecular level interaction between apoptosis and long-term depression [8].

The chronic unpredictable mild stress (CUMS) model mimics human depression in its cause and pathogenesis; therefore, it is considered a reliable and valid model to study the action of antidepressant drugs on animals [10]. In the CUMS model, animals are exposed to a series of minor-intensity stressors at unpredictable times for several weeks. Animals suffer from a series of behavioral, neural, biochemical, cellular, and endocrinal changes, which resemble those that occur in human cases of major chronic depressive disorder [11].

Conventional pharmacological and psychological antidepressant therapies are considered the primary line of treatment of depression [12]. One of the new generation antidepressants is fluoxetine which is a selective serotonin reuptake inhibitor (SSRI) [13]. Currently, the use of the available antidepressants is limited due to several factors such as the delayed onset of the antidepressant effect [14], the associated pharmacological side effects which lead to patient incompliance to therapy [15], drug resistance, and finally lack of response to the drug [16].

On the opponent side, utilization of alternative and complementary medicine is considered a second promising treatment option [17]. Functional foods or herbal medicines are intensively studied for their therapeutic potential against depression as most of the drugs in the market are expensive and cause many side effects [18]. Pumpkin, a member of the Cucubitaceae family, is used as an herbal medicine [19]. Pumpkin was proved to have many biological effects including antifatigue, antioxidant, anti-inflammatory, anticarcinogenic, antimicrobial, and antiobesity activities [20, 21]. *L. Cucurbita pepo* is one of the three commonest Cucubitaceae species cultivated worldwide [22].

Among the common nutritional deficiencies observed in patients with mental disorders are the amino acids that are the precursors of neurotransmitters [23]. Pumpkin contains the essential amino acid L-tryptophan. The latter as well as its intermediate metabolite 5-hydroxytryptophan (5-HTP) enter in the formation of the neurotransmitter serotonin; therefore, they are endorsed for treatment of depression [23, 24].

In a previous study, aqueous extract of *Cucurbita pepo* seeds was described as a potential source of natural psychotherapeutic agents against depression because it possessed a significant antioxidant and antidepressant activity [25]. Pumpkin (*Cucurbita maxima*) seeds, either raw or processed, were also reported to reduce the depressive symptoms in rats in a comparable manner to that of imipramine [26]. In a more recent study, LaChance and Ramsey summarized the antidepressant foods and reported that pumpkin seeds had an antidepressant food score of 47% [27]. In addition, Sweetme Sweet Pumpkin™ and its active compound, *β*-carotene, was proved to have an antidepressant-like effect [28]. The authors of this study called for more investigation to confirm and explore the antidepressant effect induced by pumpkin before clinical application in humans. Therefore, this study was designed to investigate the antidepressant potential of *L. Cucurbita pepo*, in an animal model of CUMS, and explore its effect on the hippocampal structure as well as its mechanism of action in comparison to fluoxetine.

## 2. Methods

The primary assessed outcome was to evaluate the antidepressant, anti-inflammatory, and antioxidant effects of sweet pumpkin, while the secondary assessed outcome was to assess the neuroprotective effect of sweet pumpkin and explore the mechanism behind it.

### 2.1. Extraction of Pumpkin (*L. Cucurbita pepo*) and Dosage

Fresh pumpkin (*L. Cucurbita pepo*) (LC) fruits were purchased from the market in Jeddah, Saudi Arabia. The fruits were identified by a botanist at the Faculty of Science, King Abdulaziz University, Jeddah.

Extraction of LC was done according to the previously described method [20]. First, the seeds were removed from the fruit; then, the skin and pulp were dried by a lyophilize machine freeze-drier (FD5508; ILShinBase Co., Ltd., Korea) and crushed by a grinding electrical machine. The powder was passed through a 40-mesh sieve to get the fine powder and stored in an airtight container.

The dried powder (50 g) was mixed with 450 mL ethanol (80%) for 1 day at 37°C temperature, left in a shaker machine (JSSI-100T; JS Research Inc., Compact Shaking Incubator, Korea) for another day, and then filtered with cotton and filter paper. This extraction process was repeated twice, and the excess solvent was evaporated under reduced pressure using a rotary vacuum evaporator (HS-2005S; HAHNSHIN Scientific Co., Ltd., Korea) at 25°C. The extract was left at a fume hood to allow extra evaporation of ethanol; then, the extract was dried in a freeze-drier machine (FD5508; ILShinBase Co., Ltd., Korea). The extract was dissolved in “0.03% sodium carboxymethyl cellulose (CMC-Na)” to be administrated orally using gavage (100 mg/kg) for two weeks [20].

Fluoxetine (Misr Co. for pharm. ind. S.A.E.) was used for pharmacological validation of LC extract after being dissolved in 0.03% CMC-Na and was orally administrated using gavage (20 mg/kg) for two weeks [29].

### 2.2. Gas Chromatography and Mass Spectrometer (GC-MS) Analysis of the LC Extract

The components of LC extract were identified using the Trace GC-TSQ Mass Spectrometer (Thermo Scientific, Austin, TX, USA) with a direct capillary column TR–5MS (30 m 0.25 *μ*m 9 0.25 *μ*m film thickness).

### 2.3. Experimental Groups

Forty male albino rats weighing from 150 to 200 g (2-3 months) were obtained from the animal house of King Fahed Medical Research Center at King Abdulaziz University, Jeddah, Kingdom of Saudi Arabia. Only male rats were included in the study to nullify the effect of gender as a confounder. The rats were left to acclimatize in the laboratory conditions for one week before starting the experiment. Ten rats were assigned as a negative control group and were left unexposed to stress. The other thirty rats were subjected to the procedure of chronic unpredictable mild stress (CUMS) that included exposing rats to various stressors at different times during the day for four weeks in order to avoid acclimatization with stress as was previously mentioned [30, 31].

Rats exposed to CUMS were assigned at a simple random method into 3 groups (*n* = 10 each). The positive control group (CUMS) was given 0.03% CMC-Na, the vehicle, by gavage for two weeks. The fluoxetine-treated group (CUMS+Flu) received fluoxetine for two weeks. The pumpkin extract-treated group (CUMS+pump) received LC for two weeks. All treatments started from the 15^th^ day and continued to the 28^th^ day of exposure to CUMS. All rats in all groups were included in the study as there were no criteria for inclusion or exclusion of rats.

### 2.4. Assessment of Behavioral Changes

In order to confirm the effect of CUMS on the rats, a forced swim test (FST) and elevated plus maze (EPM) were conducted for all rats, at the end of the experiment, as was previously described [31, 32]. During the FST, the rat was left to swim in glassy cylindrical container water at 25 ± 2°C. The rats were observed by a technician for 6 minutes. The total time spent by the rat immobile during the 6 minutes was determined in seconds. Immobility was identified as “the cessation of limb movement, except for the minor movement necessary to keep the rat floating.” In EPM, the number of closed arm entries during 6 minutes and the time spent by each rat in the open arm were assessed in seconds.

### 2.5. Assessment of Corticosterone Level in Serum

After finishing the behavior tests, rats were anesthetized with 4% isoflurane (SEDICO Pharmaceuticals Company, Cairo, Egypt) in 100% oxygen then euthanized by cervical dislocation. Blood samples were obtained from the heart for biochemical assessment. Centrifugation was performed at 3000 rpm for 15 min at 4°C to obtain the serum that was kept at −18°C. The corticosterone level (ALPCO Diagnostics, Orangeburg, NY, USA) was assessed in the serum using ELISA kits as was described in the manufacturer's instructions.

### 2.6. Assessment of TNF-*α* and IL-6 in the Serum

Sera were used to assess TNF-*α* and IL-6 (quantakin R&D system, USA Kit) using ELISA as was described in the manufacturer instructions. The optical density of each sample was measured in duplicate with “a microplate ELISA reader set to 450 nm.”

### 2.7. Assessment of Malondialdehyde [33], Superoxide Dismutase (SOD), Glutathione Peroxidase (GPX), and Catalase (CAT) in the Serum

The level of MDA was assessed spectrophotometrically at 535 nm using the Thiobarbituric Acid Reactive Substances (TBARS) Assay Kit (Biodiagnostic; Egypt) according to the method described by Gamal et al. [34]. The activity of SOD was assessed using the SOD Assay Kit (Biodiagnostic; Egypt) according to the methods described by Packer [35].

The glutathione peroxidase kit (Randox Labs, Crumlin, UK) was utilized to assess GPX according to this method previously described [34]. The CAT activity was assessed using CAT assay kits (Biodiagnostic; Egypt) [34].

### 2.8. Assessment of Caspase-3, Ki 67, and GFAP Gene Expression Using RT-PCR

Immediately after cervical dislocation, the whole brain was carefully dissected, immersed in dried ice to be divided into left and right hemispheres, fixed in 10% neutral buffered formalin, and routinely handled to obtain paraffin blocks in the histopathology lab. In order to perform RNA extraction, about 100 mg of formalin-fixed paraffin-embedded (FFPE) sections obtained from the left brain hemisphere at the hippocampus was deparaffinized in 1 mL of xylene and further processed as was described by Pikor et al. [36].

Extraction of total RNA using TRIzol was done according to the supplier instruction (Invitrogen Life Technologies, Carlsbad, CA, USA). A NanoDrop 2000 Spectrophotometer (Thermo Scientific, USA) was used to measure the concentration of RNA. Reverse transcription was done by oligo-dT primers (Bioneer Inc., Daejeon, Republic of Korea) in a 20-ll reaction including 5 ll RNA as was previously described [37]. Caspase-3 (forward 5′-*TGTATGCTTACTCTACCGCACCCG*-3′, reverse 5′-*GCGCAAAGTGACTGGATGAACC*-3′), Ki 67 (forward 5′-AAGAAGAGCCCACAGCACAGAGAA-3′, reverse 5′-AAGAAGAGCCCACAGCACAGAGAA 3′), GFAP (forward 5′-CAAGCCAGACCTCACAGCG-3′, reverse 5′-GGTGTCCAGGCTGG-TTTCTC-3′), and *β*-actin (forward 5′-TCTGGCACCACA CCTTCTA-3′, reverse 5′-GGCATACAGGGACAGCAC-3′) were used, in this study.

PCR amplification was done in a thermocycler (manufactured by Labnet International Inc.). Using a comparative Ct method, normalization of current results to *β*-actin, the reference gene, was done. Ct values were used to estimate the gene/*β*-actin ratio, with a value of 1.0 used as the control (calibrator). The normalized expression ratio was calculated using the 2−*ΔΔ*Ct. The level of mRNA was presented as a ratio or percent to that of corresponding *β*-actin.

### 2.9. Histological Techniques

The right hemisphere of the brain was processed into paraffin blocks and sectioned at 4 *μ* thickness then stained with Haematoxylin and Eosin (H&E). In addition, another set of paraffin sections was immunohistochemically stained using the streptavidin-biotin-peroxidase technique. The antibodies used included anti-glial fibrillary acidic protein (GFAP) (Dako Cytomation, USA, at the dilution 1 : 1000), a specific marker of astrocytes, as well as anti-caspase-3 (Santa Cruz Biotechnology, USA, at the dilution of 1 : 1000) to detect apoptosis.

The nuclei were counterstained with Haematoxylin, and brown cytoplasmic staining was considered a positive reaction. Olympus Microscope BX-51 (Olympus) with a digital camera connected to a computer was utilized by a histopathologist blind to the study groups for examining and photographing the histological slides. Pro Plus image analysis software was used for semiquantitative analysis of antibody immunoreactivity. The positive cells were counted per 1.0 mm^2^ of the area of hippocampal CA3 subregion and dentate nucleus as was previously described [38]. At least five fields from each slide were examined, and the mean was calculated for each animal.

### 2.10. Statistical Analysis

The Statistical Package for the Social Sciences (SPSS) version 16 was used to perform statistical analysis of data obtained from the behavioral, biochemical, and immunohistochemical assessment. The experimental unit in this study was a single animal. All experimental units were included in the analysis.

In order to test the assumption that the sweet pumpkin has antidepressant effect through modulating the depression-associated inflammatory and oxidative effects, the following tests of significance were done. The parametric data were compared using analysis of variance, followed by a least significant difference (LSD) post hoc test to avoid a multiple-comparison effect. When normal distribution has not existed, the nonparametric Mann-Whitney *t*-test was used. The sample size was determined a priori using power analysis. *p* value < 0.05 is considered significant.

## 3. Results

### 3.1. The Constituents of Pumpkin Extract

**Figure 1:**
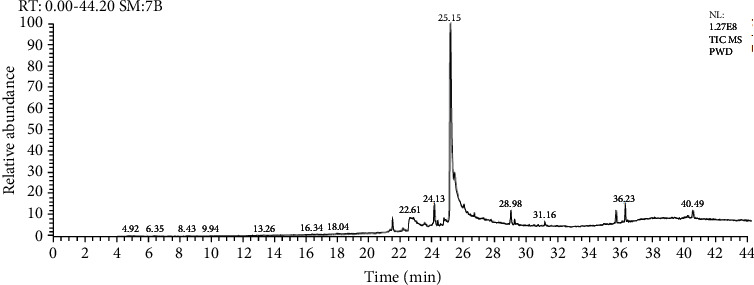
Fingerprint of *L. Cucurbita pepo* extract.

The identified chemical composition of *L. Cucurbita pepo* extract is presented in Table 1 (Figure 1). The main chemical components included were oleic acid (56.59%), hexadecanoic acid (8.90%), 10-octadecenoic acid, methyl ester (4.82%), and others.

### 3.2. Behavioral Results

**Figure 2:**
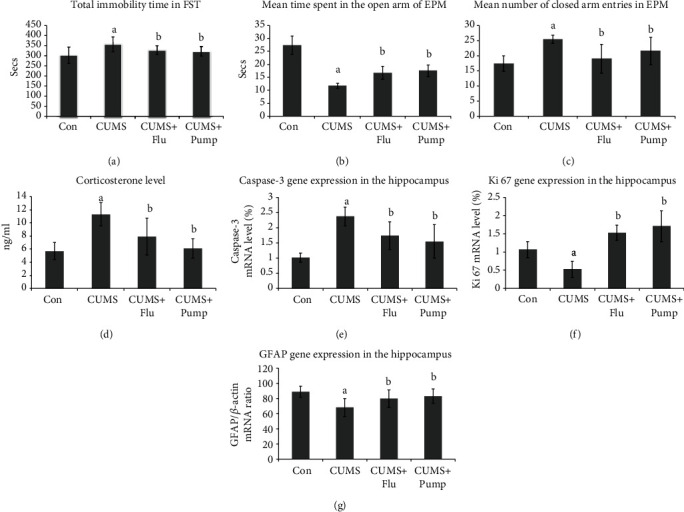
Effect of pumpkin on total immobility time in FST (a), mean time spent in the open arm of EPM (b), number of closed arm entries in EPM (c), and serum corticosterone level (d). Effect on gene expression of caspase-3 (e), Ki 67 (f), and GFAP (g) was assessed using RT-PCR. Data are shown as the mean ± SD, *n* = 10. Comparison between groups was done using a one-way ANOVA test followed by the LSD post hoc test. (a) Significance *p* < 0.05 versus the Con group; (b) significance *p* < 0.05 versus the CUMS group. FST: forced swimming test; EPM: elevated plus maze; Con: control; CUMS: chronic unpredictable mild stress; Pump: pumpkin; Flu: fluoxetine.

Behavior assessment using FST revealed a significantly longer (*p* = 0.03) immobility time of the CUMS-exposed rats than that of the control, whereas the CUMS+Flu and CUMS+Pump groups showed a significantly shorter immobility time (*p* = 0.02, *p* = 0.04), respectively, than that of the untreated CUMS group. It was notable that there was no significant difference (*p* = 0.42) in the total immobility time between the CUMS+Flu and CUMS+Pump groups (Figure 2(a)).

When it came to the EPM test, it was found that the rats of the CUMS group spent a significantly shorter time in the open arm (*p* < 0.001) than that of the control rats, whereas those of the Flu- and Pump-treated groups spent a significantly longer time (*p* < 0.001) than that of the untreated CUMS group with no significant difference (*p* = 0.45) between these two treated groups (Figure 2(b)). The mean number of rat entries to the closed arm of the EPM was significantly higher (*p* < 0.001) in the CUMS group compared to the control group, while it was significantly lower in both Flu- and Pump-treated groups (*p* < 0.001, *p* = 0.02) compared to the untreated CUMS group with no significant difference (*p* = 0.12) between the treated groups (Figure 2(c)).

### 3.3. Biochemical Results

#### 3.3.1. Corticosterone Level in the Serum

The serum corticosterone level showed a significant increase (*p* < 0.001) in the CUMS group compared to that of the control rats, whereas those treated with Flu or Pump showed a significant reduction (*p* = 0.002, *p* < 0.001), respectively, compared to the untreated CUMS group. It was observed that there was no significant difference (*p* = 0.09) in the total corticosterone level between the CUMS+Flu and CUMS+Pump groups (Figure 2(d)).

#### 3.3.2. TNF-*α* and IL-6 Levels in the Serum

Assessment of TNF-*α* and IL-6 in the serum showed that they were significantly elevated (*p* < 0.001) in the CUMS group compared to the control, whereas they were significantly reduced (*p* < 0.001) in the Flu- as well as Pump-treated groups compared to the CUMS-exposed group (Table 1).

#### 3.3.3. MDA, SOD, GPX, and CAT Levels in the Serum

A significant increase (*p* < 0.001) was observed in the MDA level of CUMS-exposed rats compared to the control rats, whereas it showed a significant reduction (*p* = 0.001, *p* = 0.003) in the Flu- or Pump-treated groups compared to the CUMS-exposed rats, respectively (Table 2).

In contrast, levels of SOD, GPX, and CAT in the serum of rats exposed to CUMS were significantly lower (*p* < 0.001, *p* < 0.001, and *p* = 0.001) than that of the control rats. The Flu-treated group showed a nonsignificant change in the levels of SOD, GPX, and CAT, whereas the Pump-treated rat showed a significant increase in SOD, GPX, and CAT levels (*p* = 0.01, *p* = 0.003, and *p* = 0.03) compared to the rats exposed to CUMS, respectively (Table 2).

#### 3.3.4. GFAP, Ki 67, and Caspase-3 Gene Expression Level in the Hippocampus

Exposure to CUMS was found to significantly upregulate (*p* < 0.001) the level of caspase-3 gene expression in the hippocampus when compared to the control group. However, this expression level showed a significant downregulation (*p* < 0.001) in the Flu- and Pump-treated groups compared to that of the CUMS group with no significant difference (*p* = 0.29) between the two treated groups (Figure 2(e)).

It was noticed that the expression level of Ki 67 gene in the hippocampus after exposure to CUMS was significantly downregulated in the CUMS group, while treatment with Flu and Pump significantly upregulated (*p* < 0.001) it. There was no significant difference (*p* = 0.17) in Ki 67 gene expression between the groups treated with Flu and Pump (Figure 2(f)).

A similar effect was induced on the GFAP gene expression level in the hippocampus of CUMS-exposed rats as it showed a significant downregulation (*p* < 0.001) compared to the control group. However, GFAP expression was upregulated in the Flu- and Pump-treated groups (*p* = 0.01, *p* = 0.002) compared to the untreated one, respectively, with no significant difference (*p* = 0.47) between the two groups (Figure 2(g)).

### 3.4. Histopathological Assessment

**Figure 3:**
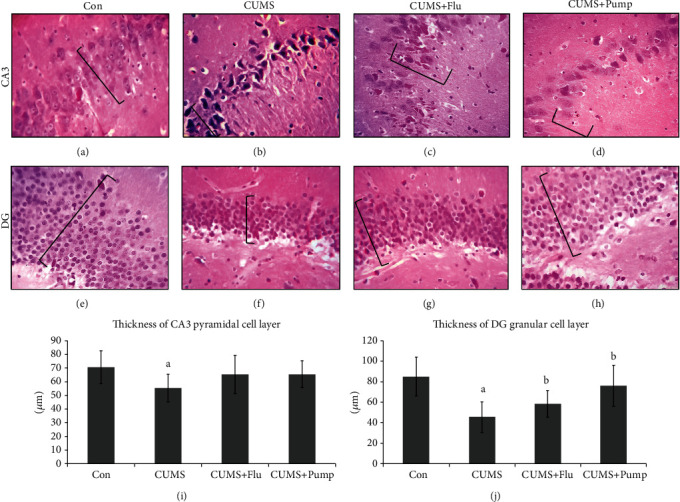
Effect of pumpkin on the histological structure of hippocampal CA3 region and dentate gyrus (DG). Photomicrographs of CA3 (a–d) and DG (e–h) region of the hippocampus. The practice indicates the thickness of pyramidal and granular cell layers (H&EX400). (i, j) Thickness of the pyramidal and granular cell layers in CA3 and DG, respectively. Data are shown as the mean ± SD, *n* = 10. Comparison between groups was done using one-way ANOVA test followed by LSD post hoc test. (a) Significance *p* < 0.05 versus the Con group; (b) significance *p* < 0.05 versus the CUMS group. Con: control; CUMS: chronic unpredictable mild stress; Pump: pumpkin; Flu: fluoxetine.

The effect of CUMS on the hippocampus was histopathologically investigated with specific emphasis on the DG and CA3 region that are controlling the mood. The CA3 region is consisting of three cell layers: the molecular, polymorphic, and pyramidal layers. The latter is formed of several layers of pyramidal nerve cells that have large open-face nuclei and basophilic cytoplasm. The hippocampus of CUMS-exposed rats showed that most of the pyramidal cells appeared degenerated as they possessed dark nuclei, and this layer was significantly thinner (*p* < 0.001) than that of the control rats. Treatment of rats exposed to CUMS with either Flu or Pump alleviated these pathological changes and increased the thickness of the pyramidal cell layer although the increase was not statistically significant (Figure 3).

The DG, of the hippocampus of control rats, is formed essentially of polygonal cells with basophilic cytoplasm and vesicular nuclei named “the granular cell.” The DG of rats exposed to CUMS showed many degenerated granular cells with dark nuclei. These degenerated cells were less frequently observed in both Flu- and Pump-treated rats. The thickness of the granular cell layer was significantly reduced (*p* < 0.001) in the hippocampus of rats exposed to CUMS when compared to the control rats. Administration of either Flu or Pump relieved these pathological changes and significantly increased (*p* = 0.02, *p* < 0.001) the thickness of the granular cell layer, respectively, compared to the untreated rats (Figure 3).

**Figure 4:**
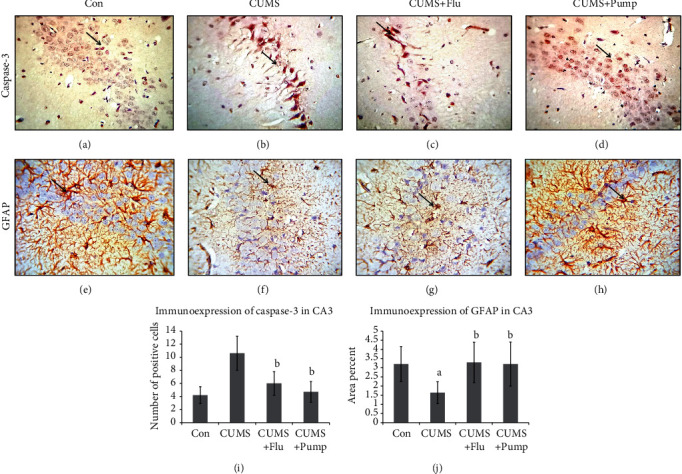
Effect of pumpkin on the immunohistochemical expression of caspase-3 (a–d) and GFAP (e, f) in CA3 region of the hippocampus. Strong positive reaction in the cells is indicated by arrow (immunohistochemical staining ×400). The percent of caspase-3 (i) and GFAP-positive cells is shown. Data are shown as the mean ± SD, *n* = 10. Comparison between groups was done using one-way ANOVA test followed by LSD post hoc test. (a) Significance *p* < 0.05 versus the Con group; (b) significance *p* < 0.05 versus the CUMS group. Con: control; CUMS: chronic unpredictable mild stress; Pump: pumpkin; Flu: fluoxetine.

**Figure 5:**
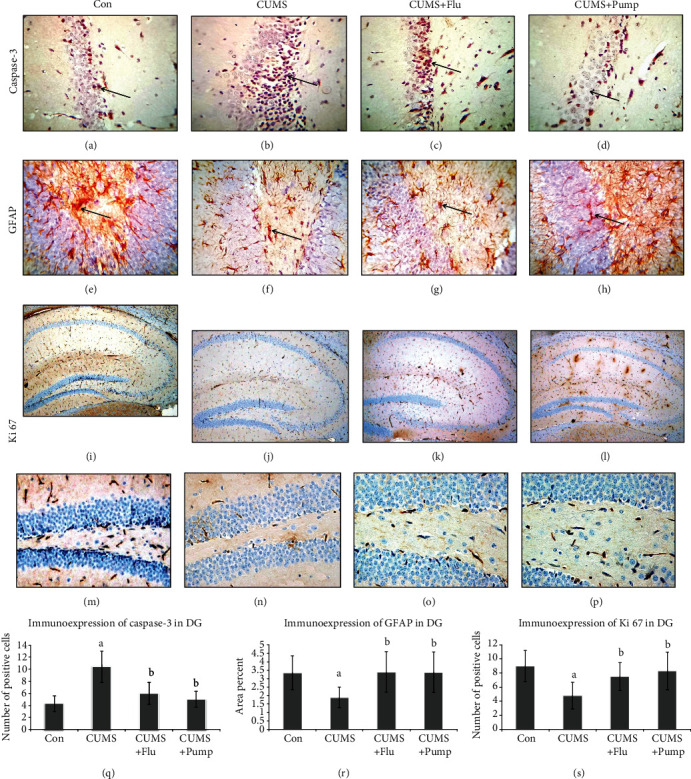
Effect of pumpkin on the immunohistochemical expression of caspase-3 (a–d), GFAP [21], and Ki 67 (P-R) in dentate gyrus (DG). Strong positive reaction in the cells is indicated by arrow (immunohistochemical staining ×400). The percent of caspase-3 (i) and GFAP-positive cells (j) is shown. Data are shown as the mean ± SD, *n* = 10. Comparison between groups was done using a one-way ANOVA test followed by LSD post hoc test. (A) Significance *p* < 0.05 versus the Con group; (B) significance *p* < 0.05 versus the CUMS group. Con: control; CUMS: chronic unpredictable mild stress; Pump: pumpkin; Flu: fluoxetine.

The apoptotic changes in the hippocampus were assessed using immunohistochemical staining with anti-caspase-3 antibodies. It was noticed that the caspase-3-positive cells were significantly more (*p* < 0.001) in CA3 and DG of the CUMS group than in the control, whereas they were significantly fewer in the Flu- (*p* = 0.01, *p* = 0.02) and Pump-treated (*p* < 0.001, *p* 0.01) groups, respectively, than the untreated group (Figures 4 and 5).

Immunohistochemical assessment of the integrity of astrocytes using anti-GFAP antibodies revealed that the number of GFAP-positive cells was significantly lower (*p* = 0.003, *p* = 0.002) in both CA3 and DG of the CUMS group when compared to the control. In contrast, the number of GFAP-positive cells was significantly increased (*p* = 0.01) in both CA3 and DG of the Pump-treated group when compared to the CUMS group (Figures 4 and 5).

## 4. Discussion

Most of the conventional antidepressant therapeutics have many side effects that limit their usage. Consequently, many strategies of alternative therapeutic are recently investigated to assess its efficacy in preventing or treating depression. In specific, the natural products that exert anti-inflammatory, antioxidant, and antifatigue effects mostly have an antidepressant-like effect [39]. Recently, pumpkin has received significant attention due to the nutritional and health benefits of the bioactive compounds present in its seeds and fruits. The extract of pumpkin was reported to have a potential antifatigue activity and can elevate exercise performance [20]. Therefore, pumpkin was selected, in this study, to investigate its ameliorative effect on the depressive status in a rat model of CUMS at the behavioral, biochemical, and histopathological level and identify the mechanism of its antidepressant effect. Willner [40] provided an updated review that confirmed the validity and reliability of chronic unpredictable mild stress as an animal model of depression that mimics human stress associated-depressive status and is considered suitable to investigate the efficacy and moods of action of substances effective in treating depression. Therefore, it was adopted in this study.

Although many studies have identified the chemical composition of seed oil of different species of pumpkin, no studies were found to identify the composition of the pumpkin fruit. In this study, the main chemical component of the extract of pumpkin fruit used was oleic acid as it represents 56.59% of its compounds. This was in agreement with many previous studies conducted on different species of pumpkin. It was reported that oleic acid represented 41.4% of the seed oil of pumpkin (*C. maxima*, var. Berrettina) [41].

For behavioral evaluation, FST and EPM were performed to confirm the CUMS-induced depressive status which was biochemically confirmed also by measuring the corticosterone level. These results were in accordance with Yankelevitch-Yahav et al. [32]. Among the biochemical changes assessed, in this study, and were found to be associated with depression are the increased serum of TNF-*α* and IL-6. The latter were reported to be high in patients with 17 scores, in the Hamilton Depression Scale [42], and in mice showing behavioral despair [43]. Elevation of these two cytokines was evident in this study.

Administration of pumpkin along with exposure to CUMS succeeded to relieve the depressive symptoms, evidenced by a reduction in the immobility time and corticosterone level, in a comparable pattern to fluoxetine as there was no significant difference between the Flu- and Pump-treated groups in these two parameters. Not only that, pumpkin reduced the elevated TNF-*α* and IL-6 in the serum of rats exposed to CUMS more than fluoxetine. Therefore, pumpkin is suggested to have an antidepressant-like effect. These findings were supported by the previous study conducted by Kim et al. [28] who found that after 4 weeks of SSP and *β*-carotene administration, the immobility time during the FST as well as the serum TNF-*α* and IL-6 significantly decreased compared to the control. These effects induced by pumpkin might be attributed to the well-known phytocompounds of pumpkin including tocopherol, carotenoid, and *β*-sitosterol that were described to have anti-inflammatory antioxidant effects [44, 45].

In this study, CUMS procedure resulted in a significant increase in serum MDA level and a significant decrease in the levels of SOD, GPX, and CAT. These findings were supported by Herken et al. [46], who found that the SOD level of the patients with major depression was significantly lower than the controls whereas it increased after 8 weeks of antidepressant treatment. These findings also were in partial agreement with what was concluded in the meta-analysis conducted by Jiménez-Fernández et al. [47] and that included 29 studies that dealt with over 2,477 patients with depression. They found that the serum level of MDA was elevated in depressed patients compared to the nondepressed persons. They added that the SOD level was increased while antioxidant-enhancing enzymes CAT and GPX were not significantly changed in depressed patients [47].

Hippocampal DG and CA3 are parts of the glutamatergic pathway which are crucial for modulation of depression response and represent a potential target for antidepressant drugs [6]. Chronic stress exposure causes atrophy of CA3 neurons in different species [48]. Recently, it was reported that impairment in the neurogenesis process in the dentate gyrus initiates depression [49]. In this study, CUMS resulted in behavioral, cytokine, and oxidative stress changes that were associated with histopathological degenerative changes and atrophy of hippocampal DG and CA3. At the cellular level, CUMS exposure induced a considerable upregulation in caspase-3 immunoexpression indicating increased apoptosis in the hippocampus, as well as reduced immunoexpression of both GFAP and Ki 67 indicating an affection of astrocytes and reduced neurogenesis in the hippocampal region, respectively. These histopathological changes were confirmed and explained at the molecular levels. It was found that exposure to CUMS markedly upregulated gene expression of hippocampal caspase-3 while it markedly downregulated both GFAP and Ki 67 gene expression. In line with that, the semiquantification of the GFAP mRNA level in the hippocampus of the CUM group was markedly decreased [50]. In addition, the downregulatory impact of CUMS on the gene expression of Ki 67, the marker of neurogenesis, was previously documented in many studies [30, 31].

The results of many previous studies are in line with our studies that showed a correlation between apoptosis and chronic stress-induced depression. Kubera et al. [3] and Liu et al. [51] confirmed that exposure to chronic stressors induced neuronal apoptosis and neuroinflammation. This apoptosis seems to be behind decreased brain volume and hippocampus size described by Cobb et al. [52]. Our findings were augmented by Si et al. [53] and Cobb et al. [54] who stated that major depressive disorder was associated with reduced astrocyte density and GFAP expression.

In this study, pumpkin as well as fluoxetine was effective in alleviating the histopathological changes induced in the hippocampus by CUMS. It reduced caspase-3 and increased GFAP expression in the hippocampal CA3 and DG. Liu et al. [50] found that the antidepressant drugs improved and alleviated the CUMS-induced effect on GFAP in rodent hippocampus. In addition, Abdel-Reheim et al. [55] reported that pumpkin seed oil reduced both physiological and pathological changes in epileptic rats. They added that pumpkin seed oil interrupted the neuronal apoptotic mechanism and reduced granular cell loss in the dentate gyrus of epileptic rats. These cytoprotective effects exerted by pumpkin on the hippocampal neurons may be attributed to its high content of phytocompounds like “*α*- and *λ*-tocopherol, *β*-carotene, *β*-cryptoxanthin, lutein, zeaxanthin, and *β*-sitosterol” [45].

In a previous study, the antidepressant mechanism of *C. pepo* in another animal model of depression was attributed to its content of flavonoids and glycosides which reach the brain tissues through the metabolizing process and subsequently protect the brain function from CNS disturbance and therefore inducing an antidepressant effect [25].

## 5. Conclusion

The results of this study revealed that the ethanolic extract of pumpkin improved depressive-like behavior, decreased serum corticosteroid and inflammatory cytokine levels, and increased the antioxidant profile in rats exposed to CUMS. It improved the hippocampus histopathological alterations observed after exposure to CUMS. This antidepressant-like effect might be mediated through downregulation of apoptosis and upregulation of neurogenesis in the hippocampus. Therefore, this study provided science-based evidence that pumpkin could be an effective antidepressant agent which is valid for testing its efficacy in treating or relieving depressive disorders in humans either alone or in combination with conventional antidepressant therapy.

## Figures and Tables

**Table 1:** The constituents of *L. Cucurbita pepo* extract.

Compound	Retention time (min)	Percentage (%)
Oleic acid	25.14	56.59
Hexadecanoic acid	22.61	8.90
10-Octadecenoic acid, methyl ester	24.13	4.82
Estra-1,3,nnnnnnnn(10)-trien-17á-ol	22.82	4.40
Sterols	—	4.02
Methyl commate	36.23	3.45
Stigmast-5-EN-3-OL (3á,24S)	35.65	3.08
Triterpene (betulin)	40.49	2.35
Linoleic acid ethyl ester	29.21	1.08
Other	—	11.31

**Table 2:** Effect of pumpkin extract on IL-6, TNF-*α*, MDA, SOD, GPX, and CAT levels in the serum of the studied groups.

Parameters	Con	CUMS	CUMS+Flu	CUMS+Pump
IL-6 (pg/mL)	25.9 ± 3.9	111.8 ± 11.7	64.5 ± 11.9	35.4 ± 6.5
		P1 < 0.001	P2 < 0.001	P2 < 0.001

TNF-*α* (pg/mL)	29.6 ± 7.8	97.2 ± 11.6	52.4 ± 11.7	42.7 ± 7.7
		P1 < 0.001	P2 < 0.001	P2 < 0.001

MDA (nmol/mL)	1.35 ± 0.14	2.23 ± 0.71	1.59 ± 0.37	1.51 ± 0.18
		P1 < 0.001	P2 = 0.001	P2 = 0.003

SOD (*μ*/mL)	18.92.9	9.9 ± 2.1	10.5 ± 3.2	13.9 ± 3.6
		P1 < 0.001	P2 = 0.62	P2 = 0.01

GPX (mu/mL)	58.6 ± 7.8	37.5 ± 4.9	40.3 ± 12.3	47.9 ± 6.9
		P1 < 0.001	P2 = 0.51	P2 = 0.003

CAT (*μ*/mL)	0.41 ± 0.09	0.27 ± 0.08	0.29 ± 0.09	0.37 ± 0.09
		P1 = 0.002	P2 = 0.61	P2 = 0.03

P1: significance versus the Con group; P2: significance versus the CUMS group.

## Data Availability

The raw data used in this study will be made available by the corresponding author upon justifiable request.

## Abbreviations

DG: Dentate gyrus
CUMS: Chronic unpredictable mild stress
SSRIs: Selective serotonin reuptake inhibitors
GC-MS: Gas chromatography and mass spectrometer
FST: Forced swim test
EPM: Elevated plus maze
MDA: Malondialdehyde
SOD: Superoxide dismutase
GPX: Glutathione peroxidase
CAT: Catalase
GFAP: Glial fibrillary acidic protein
TNF-*α*: Tumor necrosis factor-alpha.
